# Glutamine protects intestine against ischemia-reperfusion injury by alleviating endoplasmic reticulum stress induced apoptosis in rats^1^

**DOI:** 10.1590/s0102-865020200010000004

**Published:** 2020-03-09

**Authors:** Hao Xu, Guangyi Liu, Haitao Gu, Jijian Wang, Yang Li

**Affiliations:** I Master, Department of General Surgery, the People’s Hospital of Kaizhou District, Chongqing, PR China. Conception and design of the study, analysis and interpretation of data, manuscript writing, final approval.; II MD, Department of Gastrointestinal Surgery, the Second Affiliated Hospital, Chongqing Medical University, Chongqing, PR China. Conception and design of the study, analysis and interpretation of data, manuscript writing, final approval.; III MD, Department of Gastrointestinal Surgery, the Second Affiliated Hospital, Chongqing Medical University, Chongqing, PR China. Technical procedures, interpretation of data, critical revision, final approval.; IV MD, Department of Gastrointestinal Surgery, the Second Affiliated Hospital, Chongqing Medical University, Chongqing, PR China. Conception and design of the study, analysis and interpretation of data, critical revision,final approval.

**Keywords:** Glutamine, Endoplasmic Reticulum Stress, Reperfusion Injury, Ischemia, Apoptosis, Intestines, Rats

## Abstract

**Purpose:**

Glutamine, as an essential part of enteral nutrition and parenteral nutrition agent, has been widely recognized to be a kind of important intestinal mucosa protectant in clinical practice and experimental research. However, the mechanisms of its protective effects are still not fully understand. Consequently, this study aimed to explore the potential mechanism of glutamine on ischemia-reperfusion (I/R) injury induced endoplasmic reticulum (ER) stress in intestine.

**Methods:**

An experimental model of intestinal I/R in rats was established by 1 hour occlusion of the superior mesenteric artery followed by 3 hours of reperfusion. Morphologic changes of intestinal mucosa, apoptosis of epithelial cells, and expression of intestinal Grp78, Gadd153, Caspase-12, ATF4, PERK phosphorylation (P-PERK) and elF2αphosphorylation(P-elF2α) were determined.

**Results:**

After I/R, the apoptotic index of intestinal mucosa epithelial cells observably increased with notable necrosis of intestinal mucosa, and the expressions of Grp78, Gadd153, Caspase-12, ATF4, P-PERK and P-elF2αall were increased. However, treatment with glutamine could significantly relieve intestinal I/R injury and apoptosis index. Moreover, glutamine could clearly up-regulate the expression of Grp78, restrain P-PERK and P-elF2α, and reduce ATF4, Gadd153 and Caspase-12 expressions.

**Conclusion:**

Glutamine may be involved in alleviating ER stress induced intestinal mucosa cells apoptosis.

## Introduction

I/R injury refers to the restoration of blood flow to an ischemic organ and inevitably brings more severe damage than the original ischemic injury. Intestine is one of the most sensitive organs to ischemia, and its I/R injury is very common in clinical practice. It can be caused by severe trauma, blood loss or liver or small bowel transplantation. Moreover, bacterial translocation from the intestine contributes to death after multiple organ dysfunction syndrome (MODS)^1^. Therefore, attenuating intestinal I/R injury is an important clinical issue.

There is no doubt that intestinal I/R injury always accompanies cell damage, which is usually ascribed to necrosis. However, a better understanding of the mechanisms of cellular damage shows the happening of apoptosis, an autonomic ordered programmed cell death controlled by genes, which is considered to be the major mode of cell death in intestinal epithelial cells induced by I/R injury^2^. Meanwhile, there is growing evidence that ER stress plays an important role in apoptosis caused by I/R^3,4^. In other words, Intestinal I/R injury will definitely result in stimulating ER stress, increasing synthesis or accumulation of misfolded proteins. Conversely, ER stress will necessarily trigger unfolded protein response (UPR) to repair those abnormal proteins. Nevertheless, if the ER stress sustainably and seriously persists, in order to protect the ischemic tissue, the apoptotic pathway will be finally activated by UPR. And the PERK-elF2-phosphated ATF4 apoptotic pathway is the most typical performance of UPR^5^.

Lots of clinical practice and experimental research indicate that the position of glutamine as a kind of important intestinal mucosa protectant cannot be replaced, as it plays a significant role in maintaining intestinal mucosa completeness, lowering intestinal mucosa permeability, enhancing intestinal immunity, reducing bacteria translocation and preventing intestinal mucosa cell apoptosis. All these mechanisms have been confirmed by our previous study^6-8^. A recent study suggests that glutamine treatment attenuates ER stress and apoptosis in TNBS-induced colitis^9^. However, very little is known about the mechanism of glutamine in mediating pathophysiological reactions to ER stress induced by intestinal I/R injury.

In this report, we aim to use the experimental model of intestinal ischemia-reperfusion to explore whether glutamine could alleviate ER stress induced intestinal mucosa cells apoptosis.

## Methods

### Animals and experimental procedures

The experimental manipulations and surgical operations in this study were approved by the Ethical Committee of Chongqing Medical University.

In this study, adult male Sprague-Dawley rats (weigh about 250–300 g, total n=30) purchased from the Laboratory Animal Center of Chongqing Medical University, were housed and fed at a standard laboratory diet with free access to tap water. Each animal was fasted overnight before surgery. Animals were anesthetized with intraperitoneal injection of 4% chloral hydrate. The rats were randomly divided into three groups as described: sham operation group (S group), I/R group and I/R+glutamine group (I/R+Gln group), 10 rats in each group. The animals in I/R group and I/R+Gln group went through occlusion of their superior mesenteric artery for 1 hour by small atraumatic clip, and went then through reperfusion for 3 hours. S group went through the same procedure except for the occlusion*.* Besides, I/R+Gln group received Alany glutamine injection (400mg/kg/d, Huarui Pharmaceutical, jiangsu, China) intraperitoneally three days before operating. Meanwhile the other two groups were administrated with the same volume of saline. Animals were killed by overdose of chloral hydrate at the scheduled time after reperfusion, and the small intestine was removed for detection as described in our previous study.

### Morphologic observation

Morphologic changes of the intestinal mucosa were observed with ordinary microscopy and transmission electron microscopy. The length of intestinal villus was measured by the imaging system and histologic injury was evaluated and classified blindly by two independent observers using a well-known grading system graded from 0-8: grade 0, normal mucosa; grade 1, enlargement of subepithelial space at the tips of the villi with capillary congestion; grade 2, extension of the subepithelial space with a moderate lifting of the epithelial layer from the lamina propria; grade 3, epithelial lifting down the sides of villi, few tips denuded; grade 4, denuded villi with capillaries exposed; grade 5, loss of villus tissue; grade 6, crypt layer infarction; grade 7, transmucosal infarction; and grade 8, transmural infarction^10^.

### Apoptosis test

The test of apoptosis cells was applied by the terminal deoxynucleotydil transferase-mediated dexoyuridine triphosphatebiotin nick end labeling (TUNEL) technique according to the manufacturer’s protocol. Five high-power fields of each slice were randomly selected, counting the number of apoptotic cells determined by TUNEL of every 100 epithelial cells. Further, averaging those results was used as the apoptotic index.

### Western blot analysis

Protein was abstracted from frozen intestinal tissue. Extracted protein was separated by 10% sodium dodecyl sulfate–polyacrylamide gel electrophoresis (SDS-PAGE), and transferred to nitrocellulose blotting membranes (Roche, CA, USA). The membranes were incubated with primary antibodies to Tubulin (Proteintech, wuhan, china), Grp78 (Bioworld, USA), Caspase-12 (Bioss, BeiJing, China), Gadd153 (Bioworld, USA), ATF4 (Santa Cruz, USA), P-elF2 (Bioeasy, shanghai, china) and P-PERK (Santa Cruz, USA), and horseradish-peroxidase–conjugated secondary antibodies for 2 hours, then developed with SuperSignal West Pico chemiluminescent Substrate Kit (Pierce, Rockford, USA), and finally analyzed with the Bio-Image Analysis System (Syngene, Frederick, MD, USA).

### RT-PCR detection

Total RNA was extracted using a total RNA Isolation System (TaKaRa, Kyoto, Japan). Reverse transcription of total RNA and real-time PCR was performed according to the manufacturer’s instructions. The PCR was conducted for 38 cycles (94°C for 5 minutes, cycles: 94°C for 30 seconds, 58°C for 30 seconds and 72°C for 30 seconds). The primers designed and synthesized by sangon bitech (shanghai, china). β-actin, forward: 5’–CCACTGGCATCGTGATGGAC–3’, reverse: 5’–GCGGATGTCCACGTCACACT–3’, 450 bp. Grp78, forward: 5’–TATTGAAACTGTGGGAGGTGTC–3’, reverse: 5’–ATTGTTACGGTGGGCTGATTAT–3’, 114 bp.

### Statistics

Statistics were analyzed with SPSS 20.0 software (SPSS Inc., Chicago, IL, USA). Data are expressed as means ± SEM. Differences between groups were compared using analysis of variance (ANOVA) followed by LSD or Tamhane^’^s T^2^ test analysis, and *P* < 0.05 was considered statistically significant.

## Results

### The impacts of glutamine on morphologic changes

**Figure 1:**
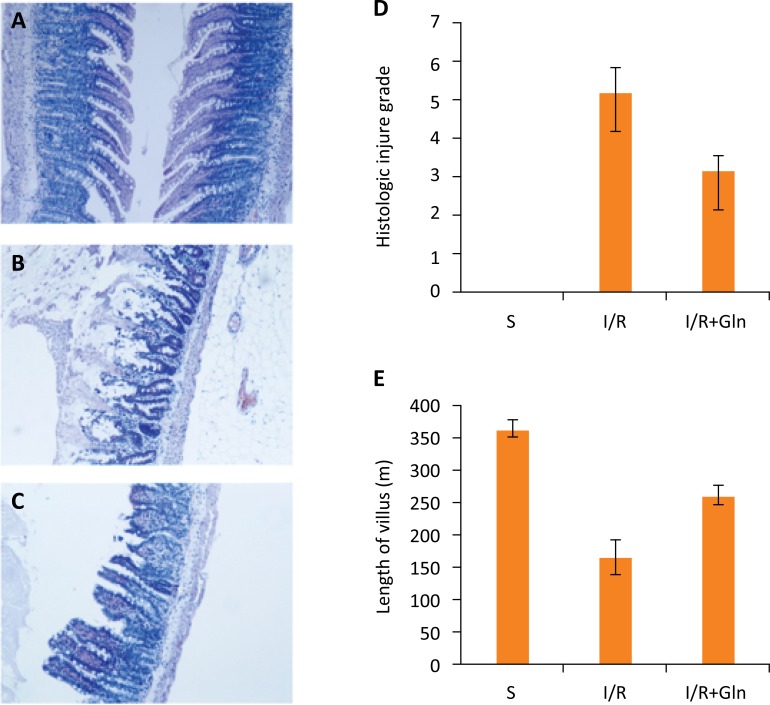
The histologic changes of intestinal mucosa under the light microscopy (×200). (A) S group, normal intestinal mucosa was seen. (B) I/R group, Intestinal mucosal villi epithelium turned into pieces, lamina propria occured bleeding ulcer, gland was seriously damaged. (C) I/R+Gln group, the edematous and hyperemic intestinal mucosa could be found. (D) Histologic injury grade of intestinal tissue. (E) Length of villus. Values are means ± SEM. Values without a common letter differ (P<0.05).

**Figure 2:**
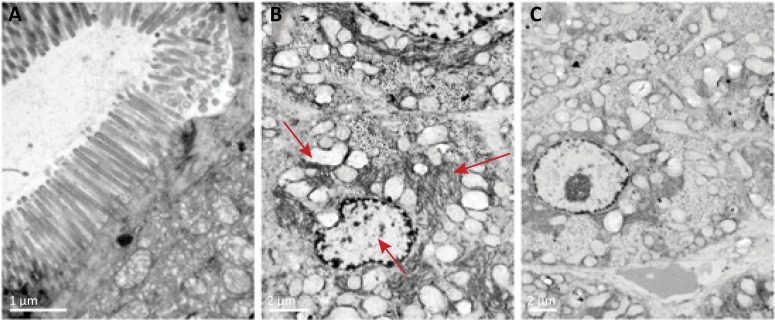
The ultrastructural changes of intestinal mucosa under the transmission electron microscopy. (A) S group, regular arrangement of cilia and normal cellular ultrastructure were seen. (B) I/R group, chromatin aggregation at the periphery of the nucleons and nuclear fragmentation, a large number of mitochondria swelling, and ER thickening were found. (C) I/R+Gln group, changes of chromatin, mitochondria swelling and ER thickening were clearly improved.

As shown in Figure 1, the histologic injury grade and reduction of villus length of I/R group were significantly higher than those in S group, whereas treatment with glutamine significantly decreased those in I/R+Gln group. In addition, the effects of glutamine on ultrastructural changes of intestinal epithelial cells are revealed in Figure 2.

### The impacts of glutamine on intestinal epithelial cells apoptosis

**Figure 3:**
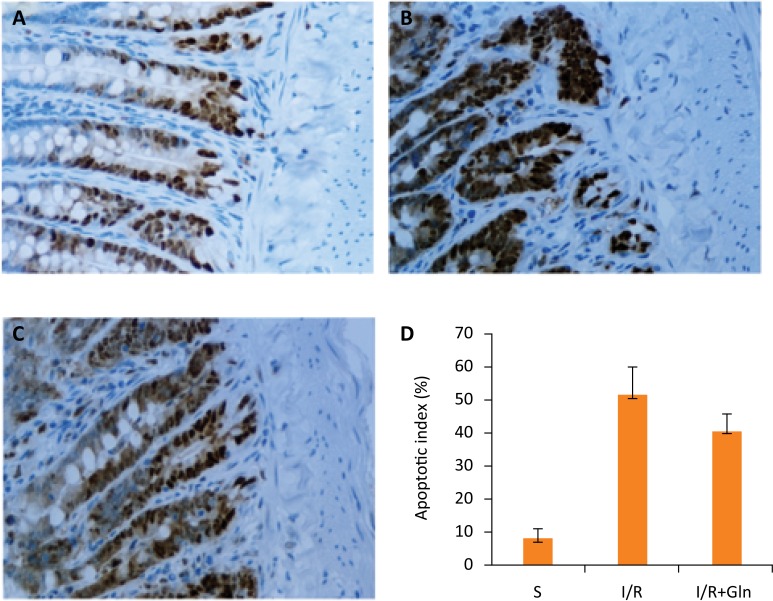
The TUNEL staining of intestinal mucosa under the light microscopy (×400). (A) S group, a few apoptotic cells were present in crypt. (B) I/R group, the TUNEL positive cells were numerous. (C) I/R+Gln group, the TUNEL positive cells clearly reduced. (D) Apoptotic index of 3 groups. Values are means ± SEM. Values without a common letter differ (P<0.05).

As shown in Figure 3, the apoptotic index of intestinal epithelial cells was significantly higher in the I/R group compared with the S group, whereas glutamine significantly decreased the apoptotic index.

### The impacts of glutamine on Grp78, Caspase-12 and Gadd153 proteins expression

**Figure 4:**
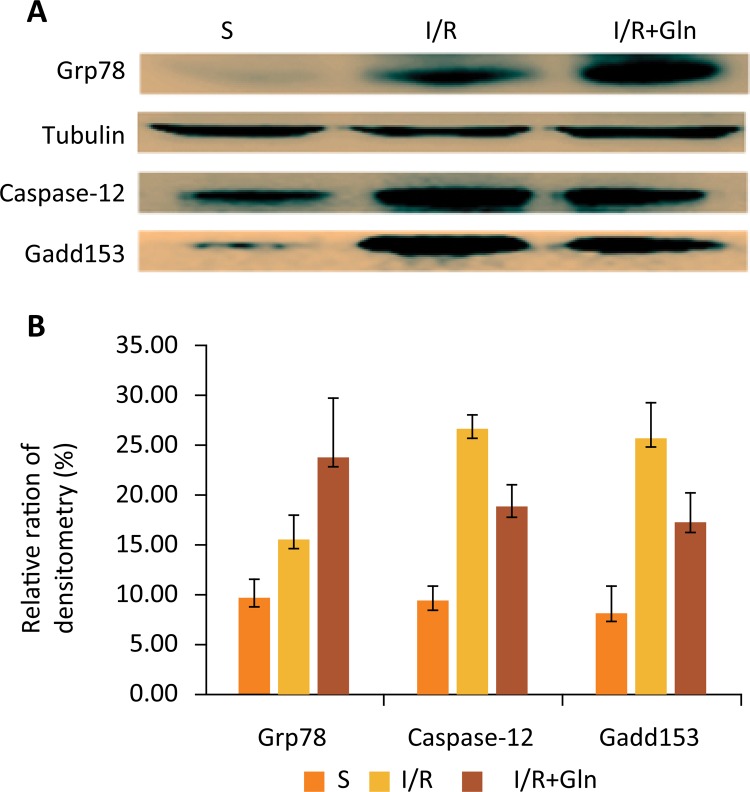
Effects of glutamine on expression of ER stress marker proteins. (A) Western blot analysis for the expression of Grp78, Caspase-12 and Gadd153 of 3 groups. (B) Quantitative detection of western blot showed that Grp78 expression distinctly increased while Caspase-12 and Gadd153 expression decreased in I/R+Gln group as compared to I/R group. Values are means ± SEM. Values without a common letter differ (P<0.05).

Grp78, Gadd153, and Caspase-12 are markers of ER stress^5,11^. So in the present study, expression of Grp78, Caspase-12 and Gadd153 in the intestinal mucosa were tested by western blot (Fig. 4). The results exposed that I/R markedly increased the ER stress marker proteins expression in intestinal mucosa in I/R group, while administration of glutamine leaded to lower expression of Gadd153 and Caspase-12 but higher Grp78 as compared to I/R group.

### The impacts of glutamine on the expression of proteins of the PERK-elF2α-ATF4-Gadd153 signaling pathway

**Figure 5:**
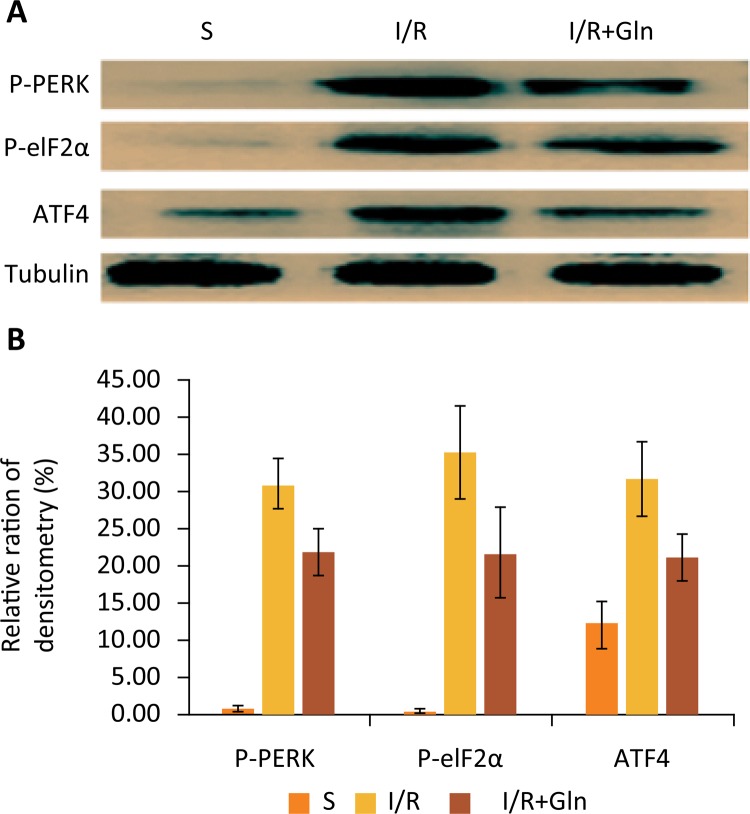
Effects of glutamine on expression of the PERK-elF2α-ATF4-Gadd153 pathway. (A) Western blot analysis for the expression of P-PERK, P-eIF2α and ATF4 of 3 groups. (B) Quantitative detection of western blot showed that the expressions of P-PERK, P-eIF2αand ATF4 were higher in I/R than in S group, while compared to I/R group, treatment with glutamine clearly decreased the expression of P-PERK, P-eIF2αand ATF4. Values are means ± SEM. Values without a common letter differ (P<0.05).

It has been demonstrated that Gadd153 expression is regulated by the PERK-elF2α-ATF4 pathway^11^. So the expression of P-PERK, P-eIF2αand ATF4 in the intestinal mucosa was detected by western blot. The results exposed that I/R observably magnified P-PERK, p-eIF2αand ATF4 expression. In contrast, glutamine precondition induced a remarkable decrease in those levels compared with the I/R group (Figure 5).

### The impacts of glutamine on Grp78 mRNA expression

**Figure 6:**
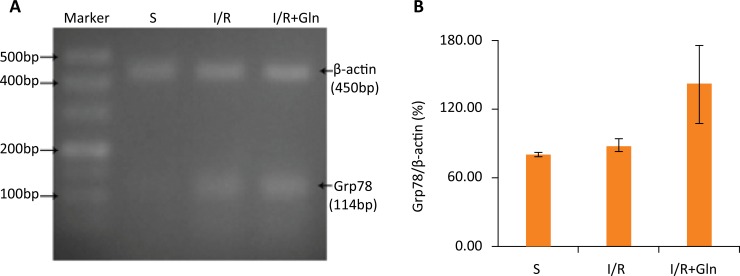
Effects of glutamine on Grp78 mRNA expression. (A) RT-PCR analysis for the expression of Grp78 mRNA. (B) Quantitative detection of RT-PCR showed that the expression of Grp78 mRNA was higher in I/R group than in S group, while compared to I/R group, glutamine treatment clearly increased the expression of Grp78 mRNA. Values are means ± SEM. Values without a common letter differ (P<0.05).

Grp78 is a member of the heat shock protein family, an important component in endoplasmic reticulum, which has been widely used as a marker for ER stress. After I/R, a significant increase in Grp78 mRNA levels was observed in the I/R group compared with the S group, while the rats treated with glutamine revealed higher in mRNA levels as compared to I/R group (Figure 6).

## Discussion

In our study, morphologic changes showed that intestinal I/R injury was remarkably ameliorated by administrating glutamine. Meanwhile, our research showed that the apoptotic index of intestinal mucosa epithelial cells observably increased with notable necrosis of intestinal mucosa (Figs. 1 to 3). In a certain sense, this also proved that the apoptosis was a major mode of cell death in the rat small intestinal epithelial cells induced by I/R injury^2^. Research found that primary intestinal mucosa epithelial cells apoptosis take place between 1 to 6 hours after reperfusion and 1 hour after ischemia^12^. Similar to that we found serious cell apoptosis occurred in 3 hours reperfusion. Above all, the intestinal mucosa epithelial cells apoptosis dramatically receded with the application of glutamine (Fig. 3).

As everyone knows, ER is the site of synthesis and folding of secretory proteins, regulating intracellular calcium homeostasis and controlling cell death signal activation^3^. At the same time, Grp78 plays an important role in maintaining ER stability and presents the basic level expression in normal physiological conditions^13^. Also, it has been demonstrated that up-regulation of Grp78 prevents tissue injury and apoptosis induced by ER stress. Otherwise, the suppression of Grp78 expression aggravated apoptosis and damage of tissue^13,14^. Because of that, Grp78 could suppress the activating of ATF6, IRE-I and PERK by bonding to those enzyme at the lumen of ER^15^. Nonetheless, prolonged ER stress, ATF6, IREI and PERK dissociating from Grp78 caused downstream transcription factors activating which controls the cellular survival pathway^16,17^. Moreover, PERK-elF2α-ATF4-Gadd153 signaling pathway is one of the most important way to activate Gadd153, a specific transcription factor located in the ER, which expresses at very low levels in normal physiological state, but is strongly induced in response to ER stress^18^. Conversely, its expression makes cells more sensitive to apoptosis^19,20^. In addition, Grp78 forms a complex with caspase-7 and caspase-12 and prevents release of caspase-12 from the ER. Caspase-12 is localized in the ER and activated by ER stress. In turn, its activation can independently induce apoptosis, and does not depend on other pathways^3,15,21^. Equally, in prolonged ER stress, the complex disrupted liberates the active Caspase-12 into the cytosol to promote cell apoptosis^3,16,17^.

As far as we know, ER stress plays a key role in the apoptosis caused by intestinal I/R injury. In this research, we observed that the expression of Grp78, Caspase-12 and Gadd153 proteins were remarkably increased after I/R (Fig. 4). This find also indicated that the UPR was initiated to confront with I/R injury. Even more important, we discovered that glutamine inhibited the increase in p-PERK, p-eIF2a, ATF4, Gadd153 and Caspase-12 after I/R in intestinal mucosa (Figs. 4 and 5). Hence, this study also showed that glutamine protects intestine against I/R injury by attenuating ER stress induced apoptosis. And such protective effect may be achieved through increasing Grp78 expression and regulating the PERK-elF2α-ATF4-Gadd153 signaling pathway.

Simultaneously, a recent study suggested that glutamine lowers the three typical markers of ER stress (Grp78, Caspase-12 and Gadd153) expression^6^. Oppositely, our study discovered that glutamine increases Grp78 expression. In our view, the result was different because of the experiment mode. Our preliminary experiments also confirmed that glutathione, the product of glutamine, could decrease apoptosis by inhibiting the NF-kB activating. Despite the controversy, the position of glutamine as a kind of important intestinal mucosa protectant is beyond dispute and contributes to alleviate ER stress induced by intestinal mucosa cells apoptosis during I/R injury. This is undeniable.

## Conclusions

Our researches strongly prove that supplement of glutamine protects vivo intestine subjected to I/R injury, which may be involved in alleviating ER stress induced by intestinal mucosa cells apoptosis. More than that, the current study can provide powerful basis for clinical application of glutamine nutrient in intestinal injury patients.
